# The Landscape of Accessible Chromatin during Yak Adipocyte Differentiation

**DOI:** 10.3390/ijms23179960

**Published:** 2022-09-01

**Authors:** Zhilong Zhang, Yongfeng Zhang, Qi Bao, Yarong Gu, Chunnian Liang, Min Chu, Xian Guo, Pengjia Bao, Ping Yan

**Affiliations:** 1 College of Animal Science and Technology, Gansu Agricultural University, Lanzhou 730070, China; 2 Key Laboratory of Yak Breeding Engineering Gansu Province, Lanzhou Institute of Husbandry and Pharmaceutical Sciences, Chinese Academy of Agricultural Sciences, Lanzhou 730050, China; 3 Key Laboratory of Animal Genetics and Breeding on Tibetan Plateau, Ministry of Agriculture and Rural Affairs, Lanzhou 730050, China

**Keywords:** ATAC-seq, chromatin accessibility, adipogenesis, yak

## Abstract

Although significant advancement has been made in the study of adipogenesis, knowledge about how chromatin accessibility regulates yak adipogenesis is lacking. We here described genome-wide dynamic chromatin accessibility in preadipocytes and adipocytes by using the assay for transposase-accessible chromatin with high-throughput sequencing (ATAC-seq), and thus revealed the unique characteristics of open chromatin during yak adipocyte differentiation. The chromatin accessibility of preadipocytes and adipocytes exhibited a similar genomic distribution, displaying a preferential location within the intergenic region, intron, and promoter. The pathway enrichment analysis identified that genes with differential chromatin accessibility were involved in adipogenic metabolism regulation pathways, such as the peroxisome proliferator activated receptor-γ (PPAR) signaling pathway, wingless-type MMTV integration site (Wnt) signaling pathway, and extracellular matrix-receptor (ECM–receptor) interaction. Integration of ATAC-seq and mRNA-seq revealed that genes with a high expression were associated with high levels of chromatin accessibility, especially within 1 kb upstream and downstream of the transcription start site. In addition, we identified a series of transcription factors (TFs) related to adipogenesis and created the TF regulatory network, providing the possible interactions between TFs during yak adipogenesis. This study is crucial for advancing the understanding of transcriptional regulatory mechanisms of adipogenesis and provides valuable information for understanding the adaptation of plateau species to high-altitude environments by maintaining whole body homeostasis through fat metabolism.

## 1. Introduction

Adipose tissue is a vital endocrine organ for energy storage and metabolism in animals, and the growth of this tissue refers to the increase in adipocyte number and size [1]. Mature adipocytes differentiate from preadipocytes under the action of a series of transcription factors (TFs), fatty acid-binding proteins, and lipid-metabolizing enzymes after growth arrest. The TFs cascade occurs prior to adipocyte gene expression during adipocyte differentiation [2]. PPARγ (peroxisome proliferator-activated receptor-γ) and C/EBP (CCAAT/enhancer binding protein) have been confirmed as pioneer TFs for inducing initial adipocyte differentiation [3]. In addition, many signaling pathways, such as insulin, glucocorticoid, BMP (bone morphogenetic protein), Wnt (wingless-type MMTV integration site) and Hedgehog signaling, have been reported to promote or inhibit differentiation of preadipocytes into adipocytes [4,5,6,7,8]. Numerous studies have found the regulatory role of TFs in bovine adipocyte differentiation. For example, by studying the mechanism of TFs regulating SIRT4 (silent information regulator Protein 4) gene during bovine adipogenesis, it was found that E2F1 (E2F transcription factor-1), C/EBPβ, and HOXA5 (homeobox A5) positively regulate the expression of SIRT4, while IRF4 (interferon regulatory factor 4), PAX4 (paired box 4), and CREB1 (cAMP responsive element-binding protein 1) suppressed its expression [9]. A previous study investigated the core promoter region of the PLIN1 (perilipin1) in bovine adipogenesis and found that E2F1, PLAG1 (pleiomorphic adenoma gene 1), C/EBPβ, and SMAD3 (SMAD family member 3) can bind to this region and activate or inhibit its transcription [10]. A similar study found that KLF15 (Krüppel-like factors 15) plays a regulatory role by binding to the core promoter region of KLF3 in bovine adipogenesis [11]. However, knowledge about genome-wide TFs regulating bovine adipocyte differentiation and the interaction network between these TFs is lacking. The assay for transposase-accessible chromatin with high-throughput sequencing (ATAC-seq) is a method that can be used to find the open chromatin region, predict TFs, and map the nucleosome position [12,13]. Chromatin in mammalian cells is usually in active euchromatin and inactive heterochromatin states. When chromatin is in the active euchromatin state, open chromatin regions with DNA regulatory elements can be bound by TFs, which will then contribute to gene regulation. When chromatin is in the inactive heterochromatin state, TFs cannot bind to transcriptionally active regions of genes, which is not conducive to gene expression [14,15]. Therefore, ATAC-seq data analysis can identify genome-wide transcription regulatory elements and transcriptionally active genomic regions in specific biological processes.

The yak is a unique and rare ruminant living in the Qinghai–Tibet Plateau and adjacent regions. As an important livestock species for animal husbandry in this area, yaks provide local residents with agricultural products such as meat, milk, and hair while adapting to environmental conditions including cold, low oxygen, and high altitude and ultraviolet rays [16,17]. Because yaks graze year-round, their growth and development is restricted by the yield and quality of forage [18]. During the warm season, the plentiful and high-quality forage feed induces weight gain and fat deposition in yaks. However, during the long cold season, yaks have insufficient nutrient intake and need to consume the fat stored in the warm season to resist severe cold and maintain the normal metabolism [19,20]. This mechanism of maintaining the normal biological process and development of the body through fat metabolism on a plateau makes the yak an ideal model for studying the adaptation of plateau species. We here used ATAC-seq to identify open chromatin regions in yak preadipocytes and adipocytes. We also applied RNA-seq to detect gene expression during yak adipocyte differentiation. Our study objectives were to (i) compare chromatin accessibility and gene expression to identify differential chromatin accessibility and differentially expressed genes (DEGs) during yak adipocyte differentiation; (ii) explore the correlation between chromatin accessibility and gene expression; and (iii) predict TF regulatory networks during yak adipogenesis. Our results provide new insights into yak adipogenesis and further reveal the mechanisms through which open chromatin regulates gene expression during biological processes.

## 2. Results

### 2.1. Landscape of Chromatin Accessibility during Yak Adipocyte Differentiation

**Figure 1:**
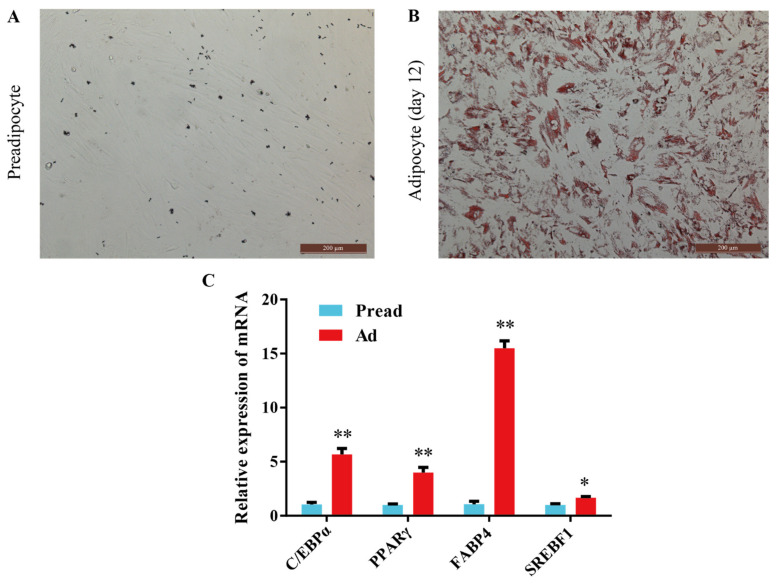
Induction of adipocyte differentiation. (**A**,**B**) Oil red O staining of preadipocytes and adipocytes. (**C**) The mRNA expression level of C/EBPα, PPARγ, FABP4 and SREBF1 between the preadipocytes and adipocytes (mean ± SEM, n = 3, * *p* < 0.05, ** *p* < 0.01).

To determine whether preadipocytes fully differentiated into adipocytes, Oil Red O staining and mRNA detection of adipogenic marker genes were performed. The results of Oil Red O staining revealed that preadipocytes were fully differentiated into mature lipid-filled adipocytes after induction with adipogenic agents for 12 days (Figure 1A,B and Figure S1). At the same time, the expression of C/EBPα, PPARγ, FABP4 (fatty acid binding protein 4), and SREBF1 (sterol regulatory element-binding factor-1) was significantly higher in adipocytes than in preadipocytes, which also supports this finding (Figure 1C).

**Figure 2:**
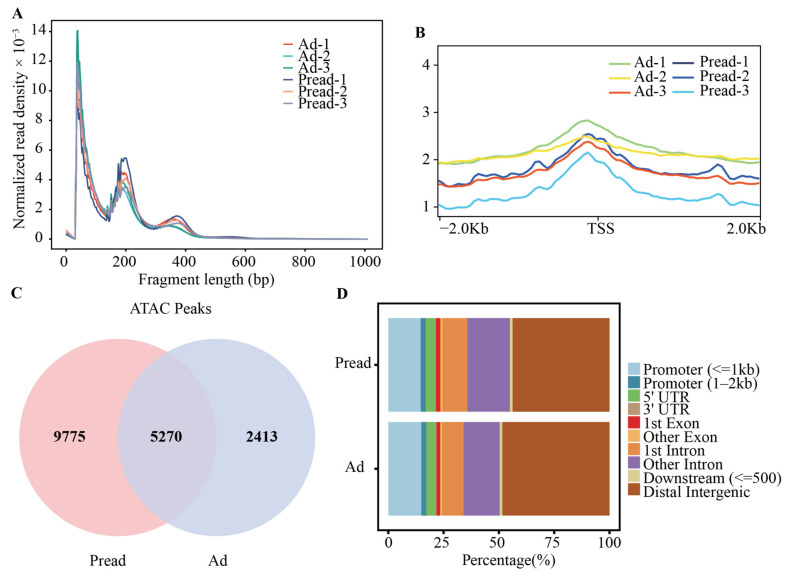
The characteristics of accessible chromatin in preadipocyte and adipocyte. (**A**) The fragment size distribution of each sample. A large proportion fragment size was less than 100 bp, which represents the regions of chromatin accessibility. The peaks at the 200 bp and 400 bp positions represent mononucleosomes and dinucleosomes. (**B**) Distribution of all reads relative to the position of TSS ± 2 Kb. The ordinate represents the ATAC-seq signal intensity. (**C**) Venn diagram showing the peak overlap between Pread and Ad groups. (**D**) The distribution of peaks among different genomic regions (promoter, 5’UTR, 3’UTR, exon, intron, downstream and distal intergenic) in Pread and Ad groups.

To explore epigenetic regulation during yak adipocyte differentiation, we used ATAC-seq to identify open chromatin regions in preadipocytes and adipocytes (three biological replicates for each cell group). An average of 167,249,845 and 159,616,847 high-quality reads were obtained from Pread (preadipocytes) and Ad (adipocytes) groups, respectively. The reads information of each ATAC-seq library before and after filtering is shown in Table S1. After the reads were aligned to LU_Bosgru_v3.0 using Bowtie2, we selected the reads aligned to unique positions for downstream analyses. Because Tn5 transposase can preferentially insert into open chromatin regions, most reads are short fragments with no or only one nucleosome. Some long fragments containing multiple nucleosomes are also present. Analysis of fragment size distribution revealed that the length of most fragments was <100 bp, indicating that the reads of each library were primarily located in the open chromatin regions (Figure 2A). Statistical analysis of all reads in the 2 kb upstream and downstream of transcription start site (TSS) by using deep Tools software showed that the highest density was observed at the TSS, suggesting that the reads were distributed according to the typical characteristics of higher eukaryotes (Figure 2B). Genome-wide peak scanning was performed using MACS software to identify regions of significant enrichment in the open chromatin. In the Pread and Ad groups, 15,045 and 7683 peaks were found, respectively. Approximately 35% of peaks detected in the Pread group were also present in the Ad group. Similarly, approximately 69% of peaks detected in the Ad group were also present in the Pread group (Figure 2C). The genomic distribution of Pread peaks showed that 43.83% of the peaks were located within the distal intergenic region, 30.48% located within the intron, 16.97% located within the promoter (2 kb upstream of the TSS), 4.13% located within 5′UTR, 2.99% located within the exon, 1.22% located within 500 bp downstream of the gene, and 0.39% located within 3′UTR. The peaks detected in the Ad group exhibited a similar genomic distribution, showing a preferential location within the distal intergenic region (48.44%), followed by the intron (26.04%), promoter (17.19%), 5′UTR (4.10%), exon (2.59%), downstream of the gene (1.17%), and 3′UTR (0.46%) (Figure 2D and Table S2).

### 2.2. Differential Chromatin Accessibility during Yak Adipocyte Differentiation

**Figure 3:**
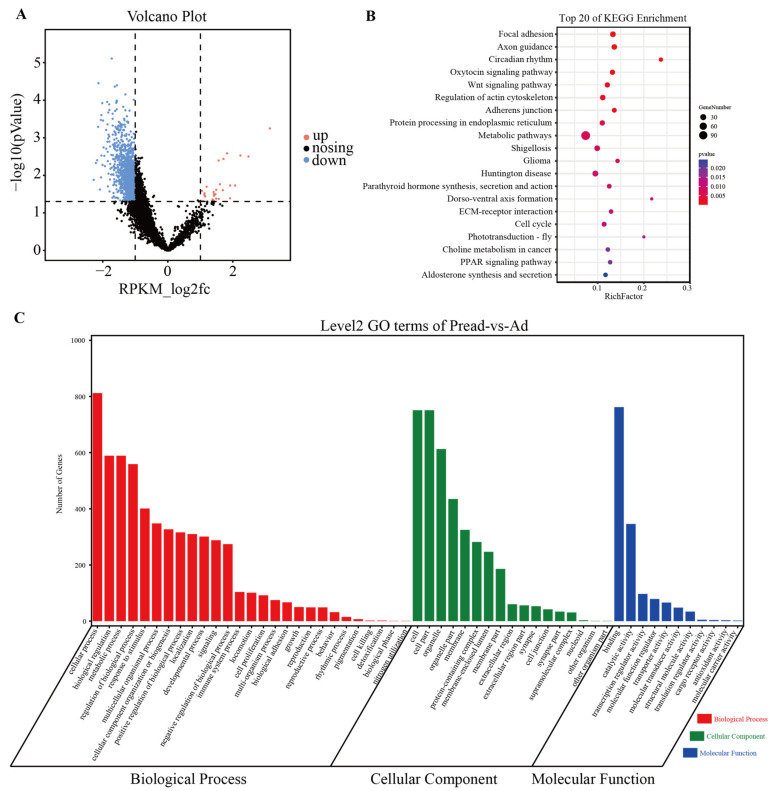
GO and KEGG pathway enrichment analysis of genes associated with differential chromatin accessibility. (**A**) Volcano plots showing differential ATAC peaks (|log2FC| > 1, *p*-value < 0.05) between Pread and Ad groups. (**B**) The top 20 KEGG pathways for the genes of differential ATAC peaks. (**C**) GO functional annotation of differential chromatin accessibility between Pread and Ad groups.

To demonstrate the role of open chromatin regions during adipocyte differentiation, we performed differential chromatin accessibility analysis between the Pread and Ad groups by using DiffBind (|log2FC| > 1, *p* < 0.05). The results revealed 1293 differential peaks, of which 1265 were downregulated and 28 were upregulated (Figure 3A, Figure S2A and Table S3). GO term analysis exhibited that these differential peaks were mainly implicated with cellular transcriptional regulatory and metabolic activities, such as binding, transcription regulator activity, and molecular transducer activity (ontology: molecular function), metabolic process, regulation of the biological process, developmental process and signaling (ontology: biological process), and cell part and protein-containing complex (ontology: cellular component) (Figure 3C and Table S4). Meanwhile, KEGG enrichment analysis revealed that differential chromatin accessibility was closely correlated to adipogenic metabolism regulation pathways including the PPAR signaling pathway, Wnt signaling pathway, regulation of actin cytoskeleton, and ECM–receptor interaction (Figure 3B and Table S5). The genetic information about these signaling pathways is presented in Figure S2B and Table S6. These results suggested that changes in gene transcription induced by altered chromatin accessibility can regulate preadipocyte differentiation into adipocytes.

### 2.3. Integration of ATAC-Seq and mRNA-Seq with Both Groups

**Figure 4:**
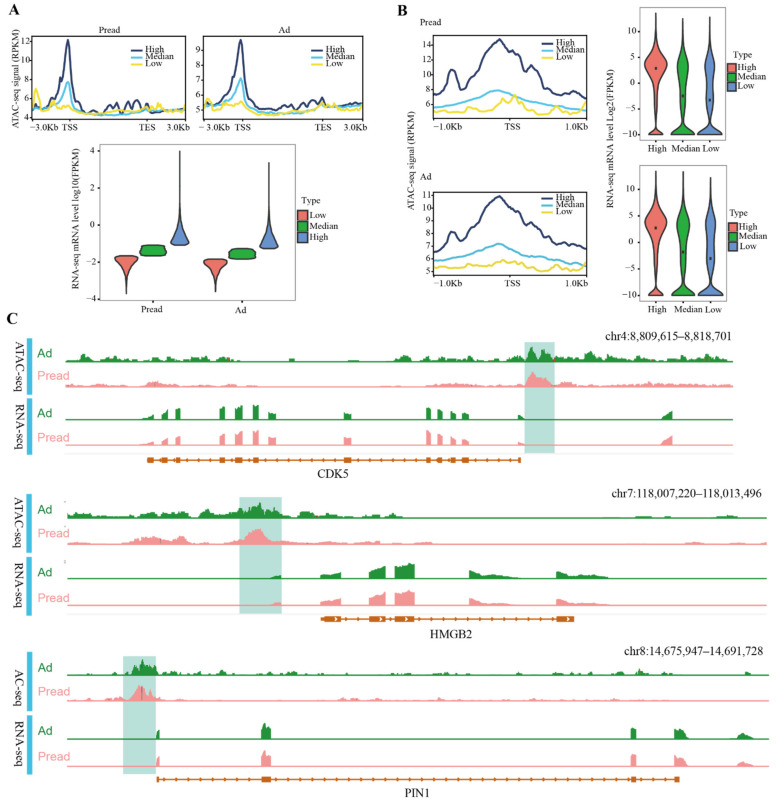
Association between ATAC signal and gene expression in Pread and Ad groups. (**A**) Association between ATAC signal within gene bodies and its 3kb upstream and downstream and gene expression. All genes were separated into three groups (high, medium, low) by expression level. Profile plots (above) display the difference in ATAC signal of each group. The horizontal axis represents different gene positions, and the vertical axis represents ATAC-seq signal intensity. Violin plot (below) show mRNA levels of high, medium and low groups divided by the gene activity value (RPKM). Plot width represents the density of repeated values in the range. (**B**) Association between ATAC signal within 1kb upstream and downstream of the TSS and gene expression. The same method is used to divide the high, medium and low groups according to RPKM and gene expression levels (FPKM). (**C**) The enrichment of ATAC-seq and RNA-seq signal near CDK5, HMGB2 and PIN1. Direction of the transcription is represented by an arrow.

mRNA-seq was used to detect gene expression changes during adipocyte differentiation. In total, 667 DEGs (|log2FC| > 1, FDR < 0.05) were identified, of which 381 genes were downregulated and 286 upregulated (Figure S3). The mRNA-seq results were validated by qRT-PCR (Figure S4). To explore the association between chromatin accessibility and gene expression in different cell types, we integrated ATAC-seq and mRNA-seq in preadipocytes and adipocytes, respectively. All genes were divided into three groups (high, medium and low) based on the gene activity value (RPKM), and the gene expression levels (FPKM) of each group were counted. The results showed that the gene expression level of the high group in Pread and Ad was higher than that of the medium and low groups (Figure 4A). This indicated that the more the open chromatin was present in preadipocytes and adipocytes, the higher the level of gene expression. Analogously, all genes were separated into three groups (high, medium and low) on the basis of the expression level, and the level of chromatin accessibility at different gene positions was counted. The results identified that the overall chromatin accessibility of the high group in Pread and Ad was higher than that of the medium and low groups. Interestingly, chromatin accessibility levels in the high and medium groups displayed obvious peaks near the TSS (Figure 4A). This suggested that the high level of chromatin accessibility located near the TSS contributes to the high gene expression level during adipocyte differentiation. To further investigate the importance of the region located near the TSS, we integrated chromatin accessibility within 1 kb upstream or downstream of the TSS and gene expression levels. We used the same method to classify chromatin accessibility and gene expression levels into three groups (high, medium and low) and analyzed their association in Pread and Ad. The results also revealed that within the 1 kb upstream and downstream of the TSS, the chromatin accessibility and gene expression levels of the high group were higher than those of the medium and low groups (Figure 4B). The high and medium groups had the highest levels of chromatin accessibility in promoter regions close to the TSS, demonstrating the vital role of these regions in gene transcriptional regulation (Figure 4B). To more intuitively show the regulation of chromatin accessibility on gene expression, we used IGV to simultaneously display the ATAC-seq and mRNA-seq signals of the fat metabolism-related genes CDK5 (cyclin-dependent kinase 5), HMGB2 (high mobility group box 2), and PIN1 (peptidylprolyl cis/trans isomerase, NIMA⁃Interacting 1). The ATAC-seq signal within promoter regions close to the TSS was obviously higher than that in the other gene regions (Figure 4C). The ATAC-seq signal levels of DEGs showed that the overall signal intensity of the Pread group was higher than that of the Ad group within the 2 kb upstream and downstream of the TSS (Figure S5A). A further analysis of DEGs found that 33 genes had differences in chromatin accessibility (Figure S5B). This indicated that differential gene expression caused by differential chromatin accessibility is a mode of gene expression regulation during adipocyte differentiation. The interaction of distal regulatory elements and proximal promoter regions is a crucial mechanism for gene regulation. Therefore, we detected the ATAC signals of the distal (>2 kb from the TSS) and proximal (TSS ± 2 kb) peaks and the gene expression level, and found that the overall signals of the distal and proximal peaks in the Pread group were higher than those in the Ad group. The gene expression also differed between the two groups (Figure S5C).

### 2.4. Prediction of TF Regulatory Networks during Yak Adipocyte Differentiation

**Figure 5:**
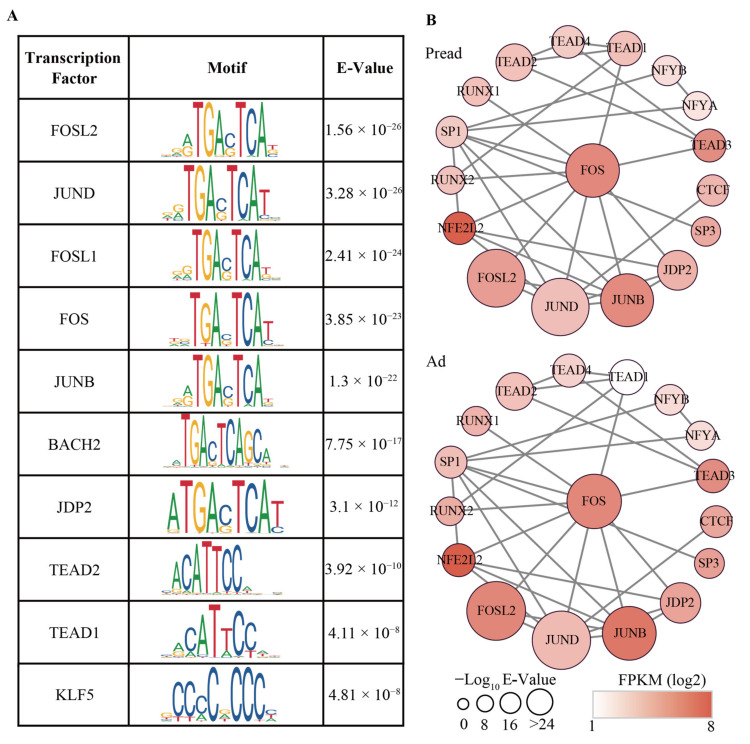
Transcriptional regulatory network during adipocyte differentiation. (**A**) Top ten transcription factors enriched in motifs of differential accessible peaks. The E-value is the adjusted *p*-value multiplied by the number of motifs in the motif file. (**B**) The interaction network between transcription factors. The size of the nodes represents the E-value of the significance. The color of the nodes represents expression levels (FPKM) of TFs.

TFs are a class of protein molecules that bind to promoters and cooperate with RNA polymerase II to initiate gene transcription. We used AME from the MEME suite (E-Value < 0.01) to identify the enriched TFs based on conservative motifs of differential peaks. A total of 26 TFs were enriched in these differential peaks, among which FOS (FBJ osteosarcoma oncogene), JUNB (jun B proto-cologene) and KLF5 (Kruppel-like factor 5) were confirmed to be related to the adipogenic metabolism regulation (Figure 5A and Table S7). The mechanism of action of TFs is usually combined with other TFs to work together. To identify the interaction between TFs, we constructed a global landscape of the TF regulatory network during yak adipocyte differentiation. The most connected TFs were FOS, JUNB, and JUND (jun D proto-cologene), and they could cooperate with many other TFs in the regulatory network. The expression levels of FOSL2, TEAD1 (TEA domain transcription factor 1) and RUNX2 were significantly different (FDR < 0.01 and |log2FC| > 0.8) between Pread and Ad groups (Figure 5B and Table S8).

## 3. Discussion

To our knowledge, this study is the first to use ATAC-seq technology to detect the landscape of chromatin accessibility during yak adipocyte differentiation. This will provide a new perspective on the molecular mechanism of adipogenesis in plateau species. Transcriptional regulatory elements of genes include enhancers, upstream activators, and proximal promoters. When the chromatin region containing transcriptional regulatory elements is in an open state, TFs can bind to the region and recruit RNA polymerase to the core promoter to initiate transcription. By contrast, condensed chromatin prevents TFs from binding to regulatory elements to silence gene expression [21]. ATAC-seq is a whole genome epigenetic detection technology that utilizes Tn5 transposase to add the adapter into open chromatin regions located primarily in promoters. The most prominent advantages of ATAC-seq are low cell input and short time of sample preparation and experiment [22]. ATAC-seq is used to study chromatin accessibility regulation during embryonic development, cell differentiation, and disease occurrence [23]. Genome-wide open chromatin detected through ATAC-seq in mouse preimplantation embryos revealed that accessible chromatin regions were widely distributed within cis-regulatory sequences whose activities diminished prior to major zygotic genome activation. Further integration of cis-regulatory sequences with single-cell transcriptomes led to the identification of essential lineage-specific regulators [24]. Use of ATAC-seq to study bovine early embryo development revealed that chromatin accessibility significantly increased during major embryonic genome activation, and its signals were strong at the TSS and transcription end sites [25]. A study on bovine myogenic differentiation found that chromatin accessibility identified through ATAC-seq exhibited dynamic changes at different time points (0, 2, and 4 days) of differentiation, indicating that open chromatin was among the epigenetic regulatory effects regulating cell differentiation [26]. Yak adipogenesis is the differentiation of preadipocytes into adipocytes; therefore, ATAC-seq can be used to identify chromatin accessibility changes during this process. Our results also suggested that open chromatin regulates yak adipogenesis, and this finding can be helpful for fat metabolism research.

We noted that the number of Pread peaks (15,045) was much higher than that of Ad peaks (7683), suggesting that ATAC-seq worked better in preadipocytes, or that differentiation would lead to reduced chromatin accessibility in yak adipocytes. The peaks of Pread and Ad groups exhibited a similar genomic distribution, demonstrating the preferential location within the distal intergenic region, intron and promoter. This distribution is consistent with the chromatin accessibility features reported in bovine myogenic differentiation, suggesting the vital role of non-promoter cis-regulatory elements in the regulation of cell differentiation [26]. Although TF-binding sites (TFBS) are mainly located in the promoter region, an increasing number of studies have shown that intron regions are also rich in TFBS. A study of regulatory elements in the human coagulation factor VIII (hFVIII) gene found that 31% of TFBS were located in intron regions and had binding preferences located far from intronic splice sites [27]. Genome-wide detection of cis-regulatory elements in early human adipogenesis revealed chromatin accessibility changes in PPARγ intron regions rich in TFBS [28]. Consequently, we inferred that the gene regulation effect of TFs binding in the intron region was also a regulatory mechanism during yak adipogenesis. Enhancer elements with sequence conservation are known to be present in intergenic regions [29]. These enhancer DNA elements are commonly recognized and bound by specific TFs to ensure transcriptional activation of target genes [30]. Meanwhile, increasing evidence suggests that enhancer–promoter interactions are also crucial for gene expression regulation [31]. Studies have shown that the majority of chromatin accessibility found during induction of T cell activation by omni ATAC-seq were located in intergenic enhancer regions [32]. A large fraction of chromatin accessibility in our study was located in intergenic regions, further suggesting that enhancers may interact with TFs and promoters to regulate gene expression during yak adipocyte differentiation.

GO terms of differential chromatin accessibility were mainly implicated with binding, transcription regulator activity, molecular transducer activity, metabolic process, regulation of biological processes, developmental processes, signaling, cell part and protein-containing complex being enriched, further illustrating that adipocyte differentiation was a complex transcriptional regulatory activity mediated by a cascade of multiple TFs. The KEGG pathway enrichment analysis found that the signaling pathways of differential chromatin accessibility was significantly associated with adipogenesis, such as the PPAR signaling pathway, Wnt signaling pathway, regulation of actin cytoskeleton, and ECM–receptor interaction. Among them, the PPAR signaling pathway, Wnt signaling pathway, and ECM–receptor interaction are the classical pathways of fat metabolism. The actin cytoskeleton is a highly dynamic structure involved in the maintenance of cell morphology and structural stability [33]. Insulin metabolism is related to actin cytoskeleton participation in the insulin signaling pathway [34]. Furthermore, investigation of the role of actin cytoskeleton in adipocyte development found that the interaction of the remodeled actin cytoskeleton and insulin signaling would affect adipocyte size [35].

Open chromatin regions can provide binding sites for TFs, thereby making initiation of target gene transcription possible. Therefore, integration of genome-wide chromatin accessibility and gene expression in specific biological processes can reveal the role that open chromatin plays in gene expression regulation. Chromatin accessibility has a significant correlation with gene expression during adipogenesis of human adipose-derived stem cells [28], bovine early embryo development [25], and bovine myogenic differentiation [26]. Our study results also revealed that genes expressed at high levels were associated with high levels of chromatin accessibility, especially within the 1 kb upstream and downstream of the TSS. Moreover, highly expressed genes exhibited the highest levels of chromatin accessibility in promoter regions close to the TSS. These results suggested that open chromatin can regulate gene expression during yak adipogenesis. We noted that only 33 DEGs identified by RNA-seq exhibited differences in chromatin accessibility, which could be explained as follows: (i) activation of target genes requires more enhancer elements in an open chromatin state; (ii) additional cofactors are required to co-ordinate TFs that bind to open chromatin regions to initiate gene transcription; and (iii) open chromatin regions may be affected by DNA methylation or other epigenetic modifications.

In the present study, differential chromatin accessibility was enriched in some crucial TFs, including FOSL2 (FOS-like antigen 2), JUND, FOS, and JUNB. FOSL2 and FOS are members of the FOS gene family. A study on the adipogenesis of deep intra-abdominal preadipocytes found that the induction of FOS protein was essential for preadipocyte differentiation into adipocytes [36]. Studies on osteoblasts from FOSL2 knockout mice in vitro found that the expression levels of adipogenic genes (C/EBPα, C/EBPβ, and PPARγ) were increased and adipocyte formation was accelerated [37]. Compared with the control mice, JUNB knockout mice exhibited reduced fat mass, higher insulin sensitivity, and increased adipose triglyceride lipase and hormone-sensitive lipase levels, demonstrating the critical regulatory role of JUNB in fat metabolism [38]. JUND can bind to the promoter of PPARγ, which contributes to the expression of FAS (fatty acid synthetase), CD36 (cluster of differentiation 36), LPL (lipoprotein lipase), and Plin5 (Perilipin 5) genes related to triglyceride synthesis, uptake, hydrolysis, and storage [39]. Based on the aforementioned findings, we infer that FOSL2, JUND, FOS, and JUNB may be involved in regulating yak adipogenesis. However, complex biological processes require multiple TFs to cooperate with each other. The members of FOS and JUN proteins can combine to form transcriptionally active complexes and exert regulatory functions during cell proliferation, differentiation, and embryonic development [40,41]. Studies have shown that the Fos–Jun complex can bind to the promoter region of the lipid-binding protein adipocyte P2 (aP2) to regulate 3T3-F442A adipocyte differentiation [42]. In our study, the core TF FOS interacted with JUNB and JUND in the TF regulatory network, further demonstrating that this interaction occurs during yak adipocyte differentiation. Interactions between other TFs were also observed in the regulatory network, and further investigation is required to clarify the mode of action of these TFs in adipogenesis.

## 4. Materials and Methods

### 4.1. Ethics Statement

All experimental procedures involved in this study were reviewed and confirmed by the Animal Administration and Ethics Committee of Lanzhou Institute of Husbandry and Pharmaceutical Sciences, Chinese Academy of Agricultural Sciences (SYXK-2014–0002).

### 4.2. Preadipocyte Isolation

Three healthy 3-day-old infant Datong yaks from Datong Yak Breeding Center (Datong County, Qinghai, China) were selected as experimental animals for this study. The collected subcutaneous adipose tissue samples were first rinsed with 0.9% NaCl and then with phosphate buffered saline (PBS) containing penicillin (200 U/mL) and streptomycin (200 U/mL). The adipose tissue was then cut into approximately 1 mm^3^ pieces in a sterile environment. Cells were isolated through type I collagenase (1 mg/mL) digestion with constant stirring for 60 min in a 37 °C in water bath. Subsequently, the digested tissue was sequentially filtered through 100 µm and 70 µm nylon mesh films, and the filtrate was centrifuged at 1400× *g* for 5 min. Cell pellets obtained through centrifugation were incubated with red blood cell lysis buffer (Beyotime, Shanghai, China) for 10 min. After washing with PBS, the mixture was centrifuged at 1400× *g* for 5 min to obtain the final cell pellets. Finally, the pellets were dissolved with DME-F12 (Hyclone, Logan, UT, USA) containing 10% fetal bovine serum (Gibco, Waltham, MA, USA), inoculated into flasks, and cultured under conditions at 37 °C with 5% CO_2_.

### 4.3. Staining of Oil Red O and Quantitative Real-Time PCR

When the density of preadipocytes reached 70–80%, the cells were induced using adipogenic agents containing 3-isobutyl-methylxanthine (0.5 mM), dexamethasone (1 μM), and insulin (10 μg/mL) (Sigma, St. Louis, MO, USA) for 2 days. The cells were then cultured with adipogenic agents including only insulin (10 μg/mL) until day 12. Finally, the adipocytes were washed three times with PBS, fixed with 4% formaldehyde crosslink for 1 h, and reacted with Oil Red O for 30 min. The stained samples were observed through light microscopy.

RNA extraction and reverse transcription of the cells were performed according to the manual of TriZol reagent (Transgen Biotch, Beijing, China) and PrimeScript™ 1st Strand cDNA Synthesis Kit (TaKaRa Bio Inc., Dalian, China), respectively. Quantitative RT-PCR was conducted on the LightCycler^®^ 96 Instrument (Roche, Basel, Switzerland) with the SYBR Green dye. Primer sequences used to determine the credibility of RNA-seq are listed in Table S9.

### 4.4. ATAC-Seq Library Preparation and Sequencing

We performed ATAC-seq using preadipocytes (n = 3) and adipocytes (n = 3). Nuclear suspensions of samples were incubated in the transposition reaction mix containing Tn5 transposase at 37 °C for 30 min. When Tn5 entered the nuclei, open chromatin regions were preferentially fragmented. Meanwhile, an adapter was added to the ends of the fragments. Immediately following transposition, the sample was purified using the QIAGEN MinElute PCR purification kit (Tiangen Biotech, Beijing, China) [43]. The libraries were sequenced using Illumina HiSeqTM 4000 by Gene Denovo Biotechnology Co. (Guangzhou, China).

### 4.5. RNA-Seq Library Preparation and Sequencing

Total RNA of preadipocytes and adipocytes was isolated using the TriZol reagent kit in accordance with the manufacturer’s manual. RNA quality was verified through RNase-free agarose gel electrophoresis and evaluated on an Agilent 2100 Bioanalyzer (Agilent Technologies, Palo Alto, CA, USA). Then, mRNA was enriched using oligo(dT) beads. Subsequently, the enriched mRNA was cut into short fragments using fragmentation buffer and reverse transcribed into cDNA by utilizing NEBNext Ultra RNA Library Prep Kit for Illumina (NEB #7530, New England Biolabs, Ipswich, MA, USA). The purified double-stranded cDNA fragments were end fixed, and a base was added. Then, the fragments were connected to Illumina sequencing adapters. The connection reaction was purified using AMPure XP beads (1.0×). Ligated fragments were subjected to size selection through polymerase chain reaction (PCR) amplification and agarose gel electrophoresis. The resulting cDNA library was sequenced using Illumina Novaseq6000 by Gene Denovo Biotechnology Co. (Guangzhou, China).

### 4.6. ATAC-Seq Data Processing and Analyses

To ensure the high quality of ATAC-seq reads, we conducted three stringent filtering standard procedures including removing reads with adapters, more than unknown nucleotides (N), and more than 50% of low-quality (Q-value ≤ 20) bases. Filtered reads of each sample were mapped to LU_Bosgru_v3.0 (Ensembl_release104) by using Bowtie2 [44] (version 2.2.8; parameters: -X 2000). Reads mapped to the + and − strands were offset by +4 bp and −5 bp, respectively. Peaks were called using MACS [45] (version 2.1.2, Yong Zhang, Boston, MA, USA) with parameters “--nomodel --shift -100 --extsize 200 -B -q 0.05”. We used ChIPseeker [46] (version v1.16.1, Guangchuang Yu, Guangzhou, China) to confirm peak-related genes and distribution of peaks in different genome regions. Significant differential peaks were identified using DiffBind [47] (version 2.8.0, Rory Stark, Cambridge, UK) with *p* < 0.05 and |log2FC| > 1. The MEME-ChIP and MEME-AME suite was used to identify the motifs. The ChIP and AME were selected to examine motifs with high credibility through peak regions and confirm the existences of any specific known motifs, respectively. Gene Ontology (GO) enrichment analysis was performed to recognize the main biological functions of peak-related genes. Kyoto Encyclopedia of Genes and Genomes (KEGG) enrichment analysis was performed to identify metabolic pathways or signal transduction pathways associated with peak-related genes. GO terms and pathways were considered to be significantly enriched if they met the threshold of *p* < 0.05.

### 4.7. RNA-Seq Data Processing and Analyses

To obtain high-quality reads, raw reads containing adapters or low-quality bases were filtered using fastp [48] (version 0.18.0, Shifu Chen, Shenzhen, China). The filtered reads were first aligned to the ribosome RNA (rRNA) database by Bowtie2 [44] (version 2.2.8, Ben Langmead, Maryland, USA). After removing rRNA reads, all clean reads were aligned to LU_Bosgru_v3.0 (Ensembl_release104) using HISAT (version 2.2.4, Daehwan Kim, Maryland, USA) [49] with “-rna-strandness RF” and other parameters set as a default. Reads aligning to each sample were assembled using StringTie (version 1.3.1, Mihaela Pertea, Maryland, USA) [50]. Meanwhile, the FPKM (fragment per kilobase of transcript per million mapped reads) value was calculated using RSEM [51] software to quantify gene abundance. DEGs between Pread and Ad groups were identified using DESeq2 (version 1.14.1, Michael I Love, Heidelberg, Germany) [52]. DEGs with false discovery rate (FDR) of <0.05 and fold change of >2 were regarded as significant DEGs.

### 4.8. Integration of ATAC-Seq and RNA-Seq

The reads belonging to each of the ATAC-seq peaks were converted to RPKM (reads per kilobase per million mapped reads). We split all genes into three groups (high, medium and low) by using a threshold value determined by dividing the gene activity value (RPKM) in three quantile groups based on their means (Hmisc::cut2 R function [53]). Then, the gene expression levels (FPKM) in each group were calculated. We used the same method to divide mRNA values (FPKM) of all genes into high, medium, and low groups and calculate RPKM in different gene positions. Integrative Genomics Viewer (IGV) (version 2.12.2, James T. Robinson, MA, USA) was applied to visualize ATAC-seq and mRNA-seq signals at the same location. The TF regulatory networks were generated using the STRING database and visualized in Cytoscape (version 3.7.1, Paul Shannon, MA, USA).

### 4.9. Statistical Analysis

Student’s *t*-test in SPSS (version 22, IBM, Chicago, IL, USA) was used to evaluate statistics. The results were displayed as mean ± SEM, and *p* < 0.05 was defined as statistically significant.

## 5. Conclusions

In summary, our study described genome-wide dynamic chromatin accessibility and gene expression during adipocyte differentiation in yaks, which are an ideal model for studying the adipogenesis of plateau species. Integration of ATAC-seq and mRNA-seq revealed that genes expressed at high levels were associated with high levels of chromatin accessibility, especially within 1 kb upstream and downstream of the TSS. Additionally, we identified a series of TFs and created the TF regulatory network, clarifying the possible interactions between TFs during yak adipogenesis. Taken together, our study sheds light on the mechanism by which open chromatin regulates gene expression during adipogenesis and provides the theoretical and material bases for research on its epigenetic roles in fat metabolism.

## Data Availability

The data were submitted to the data base of the Sequence Read Achive (SRA). The appropriate number for accession is PRJNA860771.

## Supplementary Materials

The supporting information can be downloaded at: https://www.mdpi.com/article/10.3390/ijms23179960/s1.

## Abbreviations

ATAC	seq assay for transposase-accessible chromatin with high-throughput sequencing
DEGs	differentially expressed genes
FDR	false discovery rate
FPKM	fragment per kilobase of transcript per million mapped reads
RPKM	reads per kilobase per million mapped reads
qRT	PCR real-time quantitative PCR
PCR	polymerase chain reaction
