# Supplementary material for: The Landscape of Accessible Chromatin during Yak Adipocyte Differentiation
Source: Int J Mol Sci. 2022 Sep 1;23(17):9960. doi: 10.3390/ijms23179960 (PMC9456067; doi:10.3390/ijms23179960)
Supplement: Supplementary file 1 [file ijms-23-09960-s001.zip › Supplementary Fingure S1+S2+S3+S4+S5.pdf]

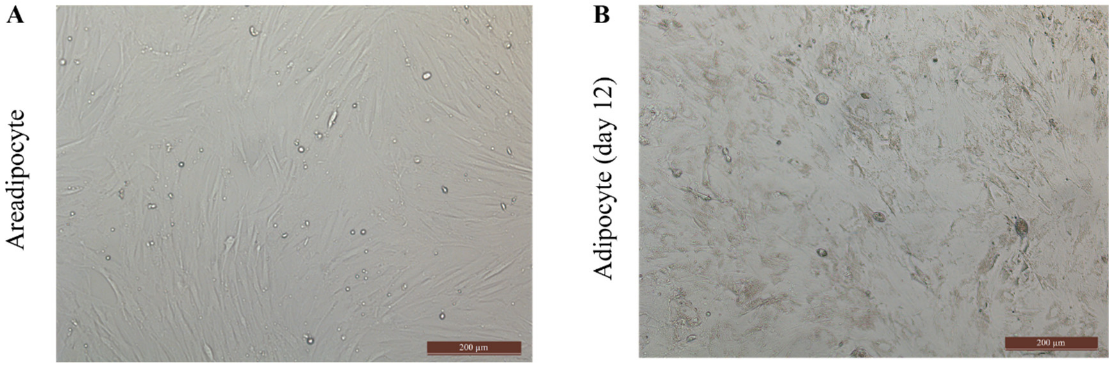

Supplementary Figure S1. Observation of cell morphology between the preadipocytes and adipocytes.

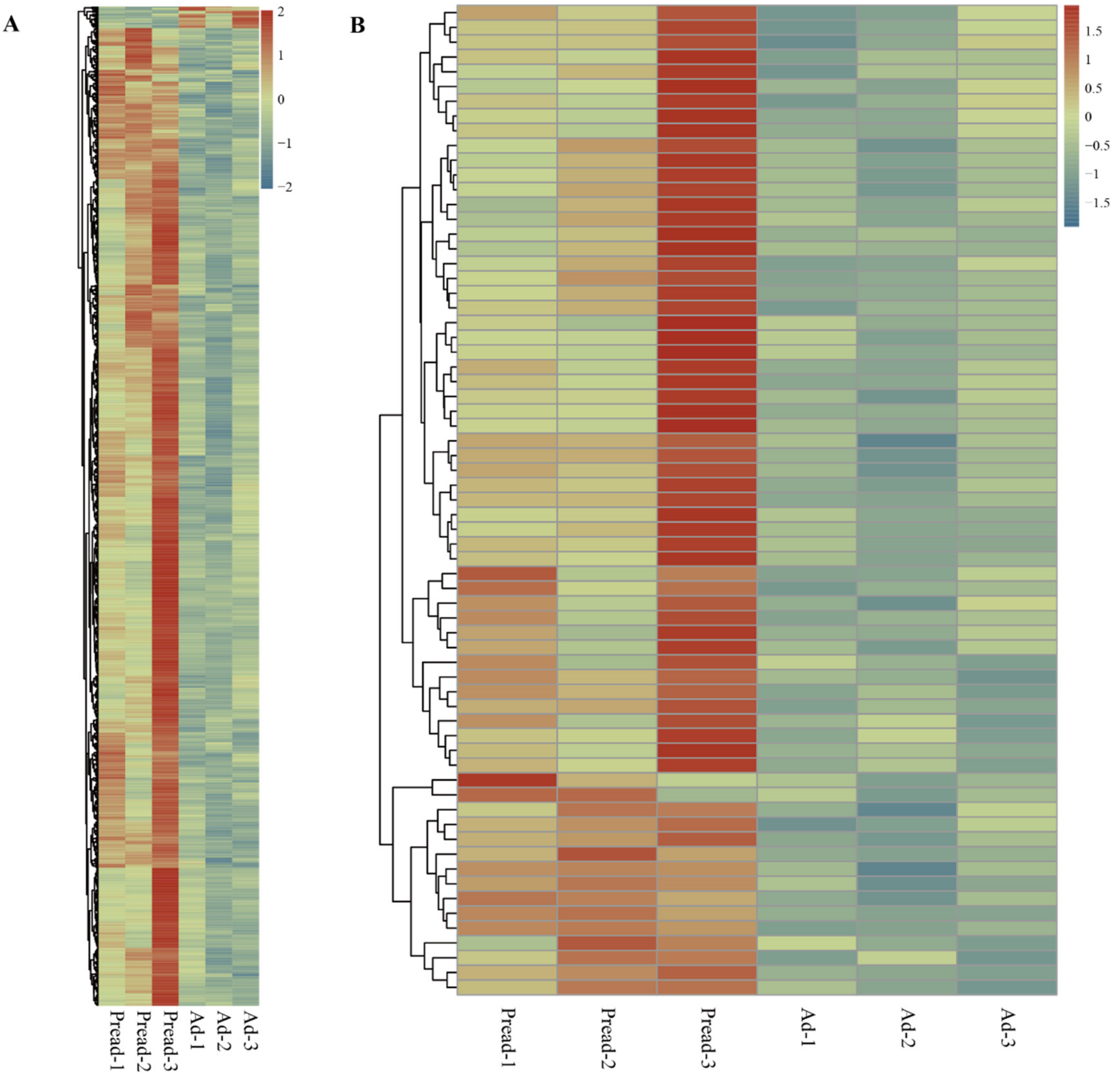

Supplementary Figure S2. Chromatin accessibility differences between Pread and Ad groups. (A) Heatmap showing 1,293 differential chromatin accessibility between preadipocyte and adipocyte ATAC-seq samples. (B) Heatmap showing 67 differential chromatin accessibility related to PPAR signaling pathway, Wnt signaling pathway, regulation of actin cytoskeleton, and ECM–receptor interaction.

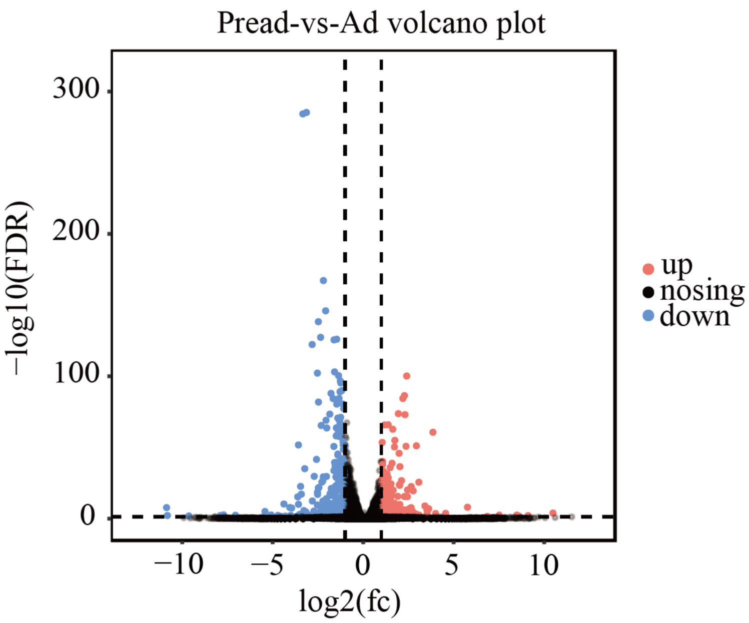

Supplementary Figure S3. Volcano plots showing differentially expressed genes from RNA-seq (|log2 FC| > 1, FDR < 0.05) between Pread and Ad groups.

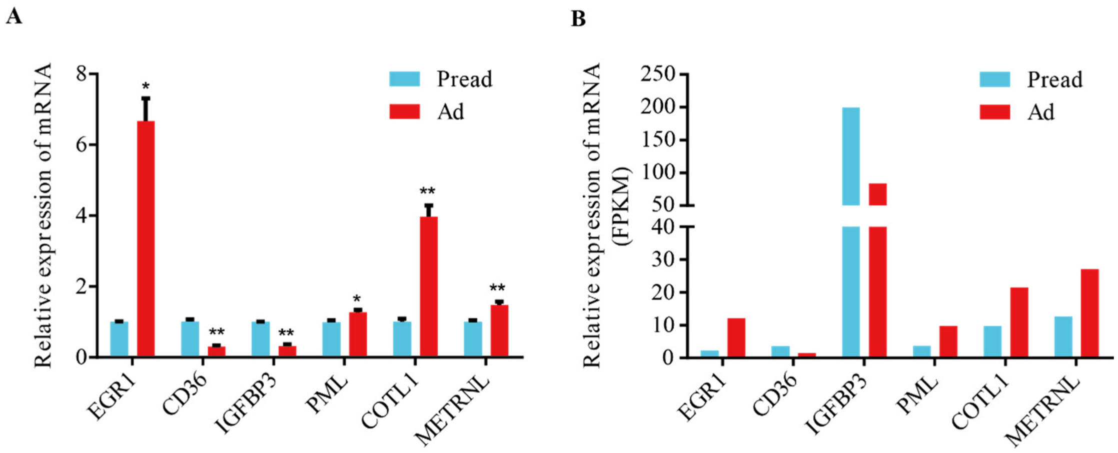

Supplementary Figure S4. Six differentially expressed genes from RNA-seq are tested by qRT-PCR in preadipocyte and adipocyte. (A) The relative mRNA levels were determined by qPCR in two groups. (B) Six genes were differentially expressed between the Pread and Ad groups based on RNA-seq data.

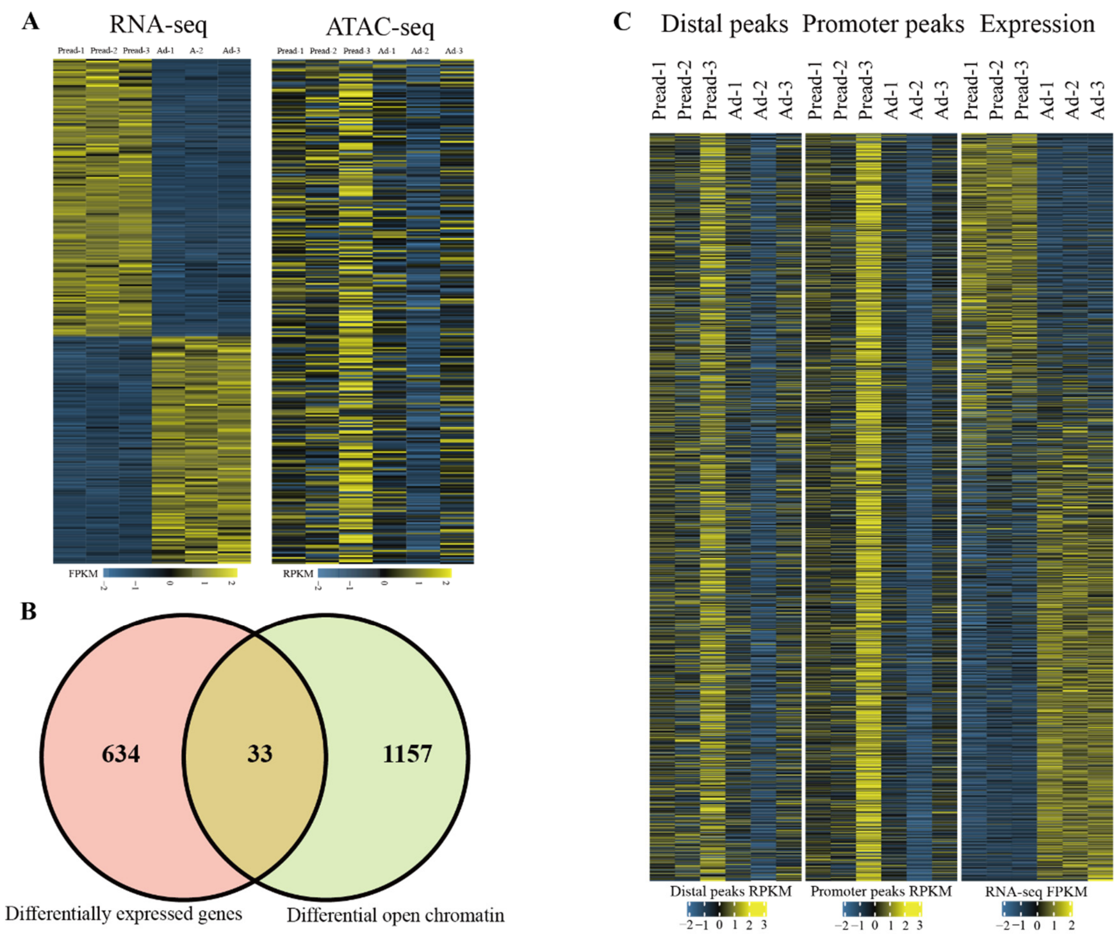

Supplementary Figure S5. Association between differential expression of genes and their chromatin openness. (A) Heat maps show expression level (FPKM) of differentially expressed genes and chromatin openness (RPKM) within their promoters (TSS ± 2kb). (B) Venn diagram showing the overlap between the differentially expressed genes of RNA-seq and differentially expressed chromatin-associated genes of ATAC-seq. (C) Heat maps showing the ATAC-seq enrichment at distal peaks (>2kb from the TSS), promoter peaks (TSS ± 2kb) and expression level of corresponding genes.
