# Supplementary material for: The Landscape of Accessible Chromatin during Yak Adipocyte Differentiation
Source: Int J Mol Sci. 2022 Sep 1;23(17):9960. doi: 10.3390/ijms23179960 (PMC9456067; doi:10.3390/ijms23179960)
Supplement: Supplementary file 1 [file ijms-23-09960-s001.zip › Supplementary Table S1+S2+S9.pdf]

Table S1 The information of filter reads

Sample	Clean_Reads_Num	HQ_Clean_Reads_Num(%)	Read_length	adapter(%)	low_quality(%)	polyA(%)	N(%)
Pread-1	191299602	190718464(99.7%)	150 + 150	63482(0.03%)	1035312(0.27%)	0(0.0%)	0(0.0%)
Pread-2	177513582	176895424(99.65%)	150 + 150	80738(0.05%)	1074836(0.3%)	0(0.0%)	4(0.0%)
Pread-3	134656446	134135648(99.61%)	150 + 150	70008(0.05%)	856180(0.32%)	0(0.0%)	45400(0.02%)
Ad-1	167789856	167236446(99.67%)	150 + 150	61744(0.04%)	983320(0.29%)	0(0.0%)	12(0.0%)
Ad-2	144408884	143912464(99.66%)	150 + 150	63914(0.04%)	816016(0.28%)	0(0.0%)	48996(0.02%)
Ad-3	168196328	167701632(99.71%)	150 + 150	68376(0.04%)	852636(0.25%)	0(0.0%)	4(0.0%)

Table S2 The distribution of peaks among different genomic regions

Area	Pread	Ad
peakCount	15045	7683
promoter	2553-16.97%	1321-17.19%
5'UTR	622-4.13%	315-4.10%
3'UTR	59-0.39%	35-0.46%
1st_exon	311-2.07%	138-1.80%
other_exon	138-0.92%	61-0.79%
1st_intron	1688-11.22%	740-9.63%
ohter_intron	2897-19.26%	1261-16.41%
downstream	183-1.22%	90-1.17%
distal_intergenic	6594-43.83%	3722-48.44%
unknown	0-0.00%	0-0.00%

Table S9 Primer information

Gene	Primer pair sequences (5'-3')	Tm (°C)
C/EBPα	F: GGCAACGACTTTGACTACCCG<br>R: CACCTTCTGCTGCGTCTCCAC	61.8
PPARγ	F: CATTTCCACTCCGCACTA<br>R: GGGATACAGGCTCCACTT	60.3
FABP4	F: CCATTAAATCCGAAAGCA<br>R: CACATTCCAGCACCATCT	59.2
SREBF1	F: TCGACTACATCCGCTTCCTT<br>R: CCACCACCTCTGGCTTCAT	59.2
EGR1	F: GAGCGATGAACGCAAGAGG<br>R: GCGATGGGTATGAGGTGGTA	61.8
CD36	F: GCAAAGAGGTCCTTACAC<br>R: ACAGCCAGGTTGAGAATG	61.8
IGFBP3	F: AAGACACGCTGAACCACCT<br>R: GGCTGCCCGTACTTATCCA	61.8
PML	F: GCCGTGAACCAGGAAAG<br>R: GGCAGGGTGGCTAAGAA	61.8
COTL1	F: CGAGCAGGGAGCGGAATA	61.8

	R: GATGAGGGCGAACTTGGA	
METRNL	F: CGACGCCACAGCAAGACA<br>R: GATGGAGCCTCGGACAACA	61.8
